# Urethral beading: A unique radiographic finding following laser lithotripsy for ureteric stent encrustation

**DOI:** 10.1016/j.radcr.2021.12.025

**Published:** 2021-12-28

**Authors:** Amber Fern Irene Matkowski

**Affiliations:** Department of General Surgery, Hereford County Hospital, Stonebow Road, Herefordshire, HR1 2BN, Wye Valley NHS Trust, UK

**Keywords:** Interventional radiology, Stent encrustation, Ureteric stent, Urology, Ureteroscopy, Lithotripsy, URSL, ureteroscopy and laser lithotripsy, CT, computerized tomography

## Abstract

We present an interesting case of ureteric stent encrustation in a 35-year-old male who was lost to follow up for 8 months during the Covid-19 pandemic. After clearing severe stent encrustation with ureteroscopy and laser lithotripsy, the patient presented with urinary retention and multiple failed catheterizations. They were found to have numerous calcified urethral fragments secondary to stent encrustation, with a unique radiographic appearance of a string of beads overlying the pubic symphysis. A new stent was inserted and the patient was lost to follow up for a further 4 months, during which time pronounced encrustation formed again.

## Introduction

Urolithiasis is the abnormal development of stones in the kidney which pass into the urinary tract, causing significant pain and potentially obstructing the outflow of urine. Failure to alleviate the obstruction can lead to permanent kidney damage and sepsis [1,2]. Surgical intervention is necessary in emergent cases and often utilizes ureteroscopy and laser lithotripsy (URSL) with stent insertion [1,3].

Ureteric stents are versatile tools in urologic surgery that provide supportive scaffolding to decompress ureteral obstruction; assist calculus expulsion; prevent occlusion; and promote tissue repair [3], [4], [5]. Stent application is considered to be a safe and simple procedure, however delayed stent removal yields complications from calcified stent encrustation that can impair a patient's quality of life, including hematuria, urosepsis, urolithogenesis, renal failure and severe flank pain [6], [7], [8], [9], [10].

A temporal relationship exists between stent indwelling time and extent of encrustation, which is influenced by the metabolic and mineral composition of urine, urine pH, bacterial colonization and stent material [7,[11], [12], [13]]. The timely removal of ureteric stents is essential to reduce the economic impact of increasingly complex management and debilitating symptoms that limit occupational capacity [14].

Prolonged indwelling typically occurs due to a deficit in patient education, patient noncompliance or logistical factors [1,3,7,15]. We present an interesting case of stent encrustation in a 35-year-old male who was lost to follow up for 8 months during the Covid-19 pandemic, who after multiple failed catheterizations was found to have multiple radio-opaque urethral stone fragments, appearing as a string of beads overlying the pubic symphysis.

## Case presentation

A 35-year-old male presented to our emergency department in July 2020 with right sided abdominal pain radiating posteriorly and moderate leukocystosis. Past medical history was unremarkable except for laparoscopic surgery for appendicectomy in 2016 and stumpitis in 2018.

Non-contrast CT of kidneys, ureter and bladder revealed a partially obstructing 11mm calculus in the right mid ureter causing mild proximal hydroureter and fullness in the pelvicalyceal system. A clinically insignificant non-obstructing caliceal calculus measuring 8.5mm in the upper pole of right kidney was also visible.

**Fig. 1:**
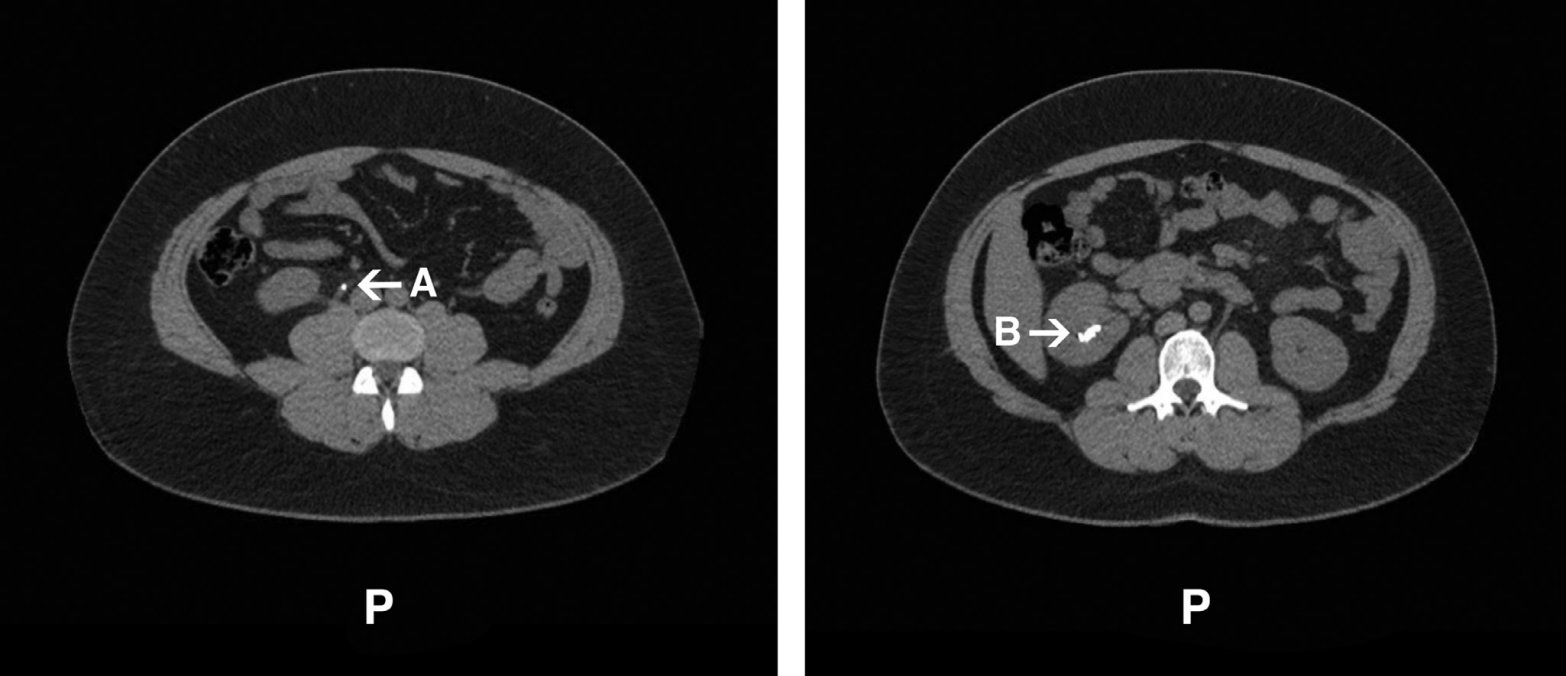
Non-contrast CT of kidneys, ureter and bladder. Transverse non-contrast CT scan of kidneys, ureter and bladder, confirming stent position, patency and successful clearance of a partially obstructive right ureteric calculus. Left: confirmed position of ureteric double-J stent in situ (A). Right: a clinically insignificant nonobstructive 8 mm caliceal calculus is seen in the upper polar region of right kidney (B).

The obstruction was treated with cystoscopy and double J stent (4.8 Fr) insertion in the right ureter; ureteroscopy failed due to tight ureter. A CT scan confirmed correct stent placement, patency, and clearance of the right ureteric calculus (Fig. 1). An outpatient appointment for right rigid and flexible ureteroscopy was arranged to follow in 6-8 weeks but due to service limitations during the pandemic, the patient was not reviewed for 8 months.

In April 2021, cystoscopy revealed a highly encrusted lower end of ureteric stent. Retrieval was difficult but eventually cleared with URSL and a new double J stent (6 Fr) was inserted. Laser lithotripsy was successfully applied to a clinically insignificant right ureteric calculus. Removal of the renal stone was not attempted due to tissue friability and arrangements were made to follow up in 6-8 weeks for flexible URSL.

Four days later, the patient presented to the emergency department with lower abdominal pain, dysuria and anuria. Multiple attempts at catheterization failed and a bladder scan demonstrated urinary retention of 804ml. A suprapubic needle was inserted to decompress the bladder and catheterization was eventually achieved with Entonox inhalation and local anesthetic with guidewire assistance.

**Fig. 2:**
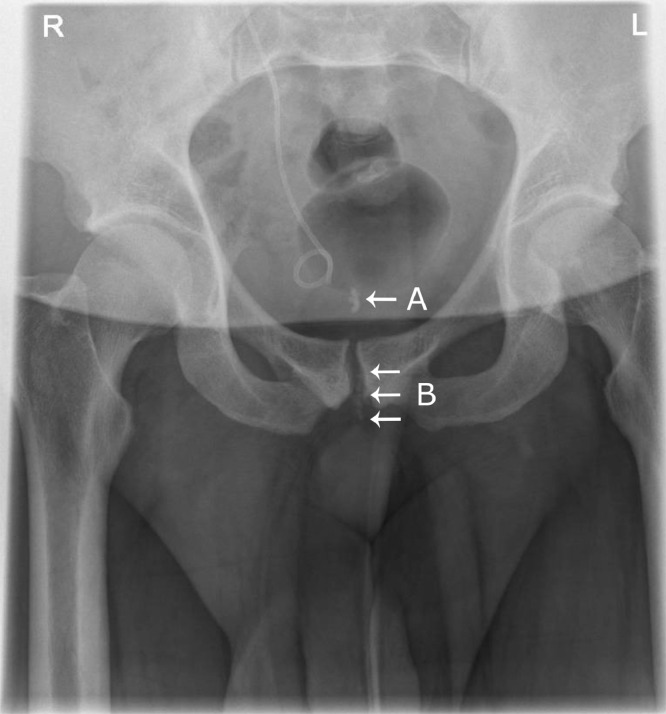
X-Ray of kidneys, ureter and bladder. Anteroposterior radiograph of kidneys, ureter and bladder demonstrates prominent calcifications in the bladder beck (A) and overlying the pubic symphysis (B), capturing a rare string of beads appearance of fragmented stones within the urethra.

Anteroposterior radiography revealed unexpected prominent calcifications overlying the pubic symphysis, capturing a rare “string of beads” appearance of fragmented stones in the urethra (Fig. 2). The patient was discharged with a urethral catheter (removed 5 days later) and antibiotics. Follow up for elective clearance of residual stent encrustation was delayed until August 2021: pronounced stent encrustation was visualized on cystoscopy and cleared with laser lithotripsy, after which the stent was easily removed and replaced. Flexible ureteroscopy showed no renal stones.

**Fig. 3:**
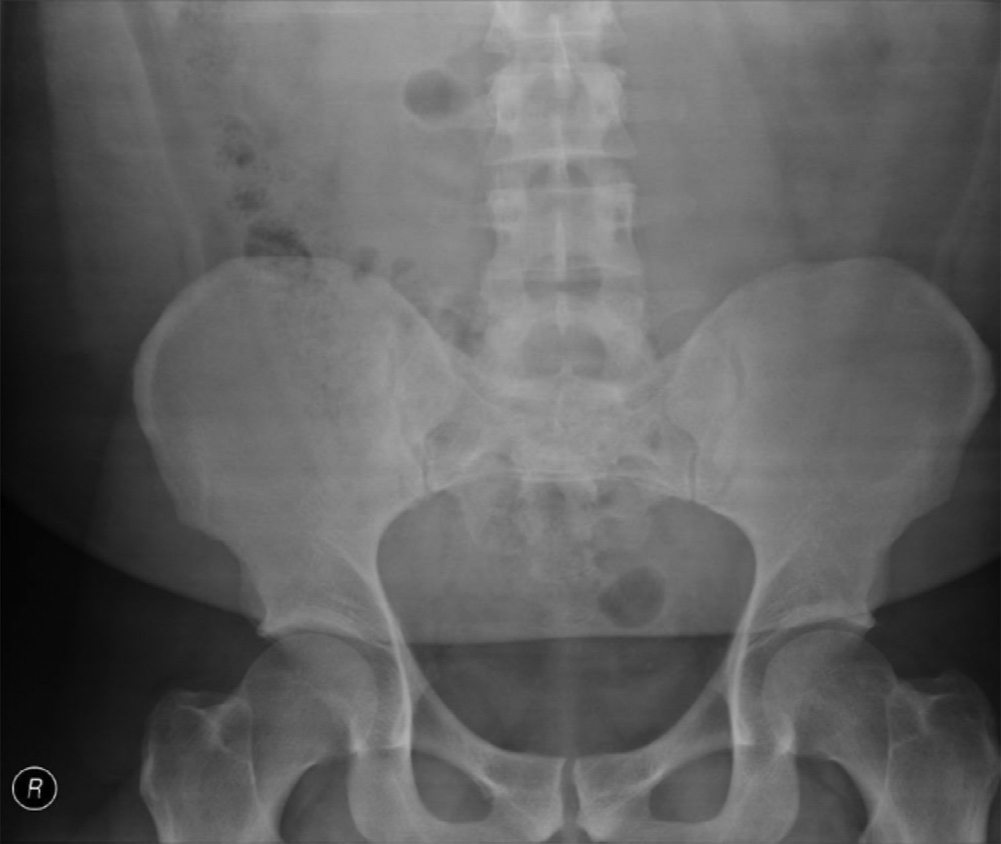

The stent was removed 2 weeks later without complications and the patient is awaiting follow up for metabolic screening.

## Discussion

Stents are accessible, relatively simple to employ and widely used for multiple urological applications, such as alleviating ureteric obstruction and assisting calculus expulsion. URSL with stent insertion is minimally invasive and can be a life-saving treatment in cases of complete or severe ureteric obstruction. But stents are not without complications: they are associated with encrustation that yields pathology similar to that being alleviated by stent insertion, including hematuria, urosepsis, renal failure and pain [6], [7], [8], [9], [10].

The process of mineralization implicated in urolithogenesis may apply to the development of stent encrustation. This involves the deposition of surface minerals that can accumulate in urine (eg, calcium and oxalate) and the presence of urease-producing organisms such as Klebsiella and Pseudomonas, which catalyze the cleavage of urea into ammonia to increase urine pH and precipitate struvite (NH_4_MgPO_4_•6H_2_O) formation [13]. Stents have been designed with different material compositions and antimicrobial surfaces to obviate mineralization but encrustation remains a significant clinical problem [4,^5^,^7^,12].

Although the pathogenesis of encrustation is multifactorial and optimal indwelling time is unclear, studies demonstrate a clear temporal relationship between duration of indwelling and incidence of encrustation [6,7]. The time-to-removal varies between centers and depends on local services, clinical context and urologists’ preference. There is also no guidance on the management of stent encrustation, which is at the discretion of the surgical team and dependent on local expertise and available facilities.

In this case, URSL was chosen as the least invasive approach to eliminate encrustation and extract the stent, although retained calcified fragments caused urinary retention requiring suprapubic catheter insertion. The patient described experiencing significant morbidity secondary to chronic pain and uncertainty while under our care, which was unfortunately impacted by service limitations during the Covid-19 pandemic. Aside from urolithiasis, the patient has no known risk factors for stent encrustation. For completeness, they will be followed up with full metabolic screening.

## Conclusion

Although widely implemented, prolonged indwelling of ureteric stents predisposes to encrustation, which is associated with significant morbidity and mortality. We document a case of severe recurrent encrustation in a patient who, after URSL treatment, was discovered to have multiple calcified fragments within the urethra, visualized radiographically with rare “string of beads” opacifications.

## Patient consent

The patient gave informed, verbal consent to the sharing of all information and images included in this case report.
